# Dental Caries Status of Institutionalized Orphan Children from Jammu and Kashmir, India

**DOI:** 10.5005/jp-journals-10005-1392

**Published:** 2016-12-05

**Authors:** Aasim F Shah, Pradeep Tangade, TL Ravishankar, Amit Tirth, Sumit Pal, Manu Batra

**Affiliations:** 1 Registrar, Department of Public Health Dentistry, Government Dental College and Hospital, Srinagar, Jammu and Kashmir, India; 2 Professor and Head, Department of Public Health Dentistry, Kothiwal Dental College and Research Centre, Moradabad, Uttar Pradesh, India; 3 Associate Professor, Department of Public Health Dentistry, Kothiwal Dental College and Research Centre, Moradabad, Uttar Pradesh, India; 4 Reader, Department of Public Health Dentistry, Kothiwal Dental College and Research Centre, Moradabad, Uttar Pradesh, India; 5 Senior Lecturer, Department of Public Health Dentistry, Kothiwal Dental College and Research Centre, Moradabad, Uttar Pradesh, India; 6 Assistant Pofessor, Department of Community Dentistry and Preventive Dentistry UCMS College of Dental Surgery, Bhairhawa, Nepal

**Keywords:** Dental caries, Jammu and Kashmir, Orphans, Prevalence, Toothbrushing.

## Abstract

**Introduction:**

It has been well documented that the absence of family support influences the general and oral health of the children. Factors that lead to the development of disease at a given point in time are likely to have their roots in a complex chain of environmental events that may have begun years before. A number of studies have examined the relationship between dental caries and material deprivation and found a positive association between them. Though orphans contribute to 2% of world’s population, literature regarding their oral health status is very scarce. This study was carried out with the aim to assess the dental caries status of institutionalized orphan children from Jammu and Kashmir.

**Materials and methods:**

A total of 1,664 children that included 1,201 boys and 463 girls from registered orphanages in the state of Jammu and Kashmir were included in the study. Written informed consent was obtained prior to the start of the study; decayed, extracted, filled teeth (deft)/ decayed, extracted, filled surface (defs) and decayed, missing, and filled teeth (DMFT)/decayed, missing, and filled surface (DMFS) indices were used to assess the caries status of primary and permanent dentition. Multiple choice, close-ended questionnaires were administered to assess the oral hygiene habits, knowledge, and dietary behavior of orphan children prior to examination. The study subjects were divided into three groups according to the age of ≤ 6, 7 to 11, and ≥12 years.

**Results:**

Results showed that caries prevalence in primary dentition was higher in subjects’ ≤6 years of age where the prevalence was 50.9%; in subjects 7 to 11 years of age, the prevalence was 25.2%. Caries prevalence in permanent dentition within the age group 7 to 11 was 69.1%, while in subjects’ ≥12 years, the prevalence was 66.2%. Use of toothbrush was the most prevalent method of cleaning the teeth in both the genders, while toothpaste was reported to be the most prevalent material to be used for tooth cleaning followed by tooth-powder. Highest caries prevalence was seen in the subjects using datun sticks as a method to clean their teeth (80.5%).

**How to cite this article:**

Shah AF, Tangade P, Ravishankar TL, Tirth A, Pal S, Batra M. Dental Caries Status of Institutionalized Orphan Children from Jammu and Kashmir, India. Int J Clin Pediatr Dent 2016;9(4):364-371.

## INTRODUCTION

Children need protection from family to build confidence to face the world and care to nurture their childhood.^1^ The United Nations Children’s Fund (UNICEF), Joint United Nations Programme on HIV and AIDS (UNAIDS), and other groups label any child that has lost one parent as an orphan. By this definition, there are currently 14 million orphans living in the world today, due to poverty, war, human immunodeficiency virus/acquired immunodeficiency syndrome, and other causes, and these contribute to 2% of the world’s population. Moreover, in the past 10 years, more than 1 million children have been separated from their families as a result of armed conflict.^2^ Out of the total orphan and street children population worldwide, the total number of socially handicapped children in India was estimated to be 2,32,46,000 in the year 2010, which accounts for 6.8% of the total child population of the country.^3^

As per the International Institute for Population Sciences and Macro International, 86.6% children in Jammu and Kashmir state live with their parents; 2.4% children have their father dead (paternal orphans); 1.6% do not have their mothers alive (maternal orphans); and 0.3% have both their parents dead (double orphans).^4^ With all these figures, it can be estimated that 4.5% of children in Jammu and Kashmir live with one or both parents dead and can be listed as orphans.^4^ In the year 2012, a “UK-based” child rights organization “Save the Children” revealed that the estimated population of orphans in Jammu and Kashmir was 2,14,000.^5^ At present, there are about 42 orphanages in Jammu and Kashmir for children 5 to 16 years of age.

It has been well documented that the absence of family support influences the general and oral health behavior and knowledge of the children. Factors that lead to the development of disease at a given point in time are likely to have their roots in a complex chain of environmental events that may have begun an year earlier.^6^

The children residing in orphanages differ from children living with parents as they are underprivileged and do not receive care as other children receive from their parents. A number of studies have examined the relationship between dental caries and material deprivation and have found a positive association between them.^7^ As known today, tooth decay is the single most common chronic childhood disease - five times more common than asthma.^8^

Though orphans contribute to 2% of the world’s population, literature regarding their oral health status is very scarce. To the fullest of our knowledge, there has been no previous study addressing the issue of oral health of orphans in the state of Jammu and Kashmir, and the previous conditions of deteriorated political peace in the region have also been a big roadblock in the path of higher researches. This study was carried out with the aim to assess the dental caries status of institutionalized orphan children from Jammu and Kashmir.

## MATERIALS AND METHODS

A descriptive cross-sectional study was conducted to assess dental caries status of institutionalized orphan children residing in orphanages of Jammu and Kashmir. This study was approved by the ethical committee of Kothiwal Dental College and Research Centre, Moradabad. The present study was planned to survey all the institutionalized orphan children in the state of Jammu and Kashmir. A list of the orphanages in Jammu and Kashmir state was acquired from the Department of Social Welfare, Jammu and Kashmir. A total of 42 orphanages were registered with the Department of Social welfare, out of which 17 orphanages were run by the state government, while 25 were run by private organizations or nongovernment organizations. The study had a total population of 1,664 children, which included 1,201 boys and 463 girls. The age group of the subjects present in the orphanages ranged from 4 to 13 years. The inclusion criteria were that children should be residing in institutionalized settings for minimum 3 years or more and give a positive consent. All those who were physically challenged and medically compromised or who were not willing to participate were excluded. Written informed consent was also obtained from the concerned orphanage directors/chairmen/ wardens after explaining the full study protocol to them.

Out of a total of 42 orphanages, 38 gave positive consent, while 2 had no children and thus were excluded. The total population of the orphanages that gave posi-five consent was 1,522, which included 1,108 boys and 414 girls. Out of these, 1,375 subjects (964 boys and 411 girls) who were present at the time of examination and gave positive consent and satisfied the inclusion criteria were examined. There were only a small number of children below 6 years of age who satisfied the inclusion criteria and less than 25 children above 13 years of age residing in these orphanages. Most of these were not present at the time of examination.

The examination was carried out over a period of 3 months from August 2012 to October 2012. A single examiner was calibrated at the Department of Public Health Dentistry, Kothiwal Dental College and Research Centre, Moradabad, in order to limit the intraexaminer variability. The agreement for all the assessments was in the range of 85 to 95%. These values reflected high degree of conformity in observations.

Data regarding age, gender, years of institutionaliza-tion, level of education of the subjects were obtained. decayed, extracted, filled teeth (deft)/ decayed, extracted, filled surface (defs) and decayed, missing, and filled teeth (DMFT)/decayed, missing, and filled surface (DMFS) indices were used to assess the caries status of primary and permanent dentition.^9,10^ No. 23 explorer (Shepherd’s Crook) and a mouth mirror were used for recording the caries. Multiple choice, close-ended questionnaires were administered to assess the oral hygiene habits, oral health knowledge, and dietary behavior of all orphan children prior to examination. The questions were adopted from Al-Omiri et al^11^ and were originally designed in English and then translated into the local language. Prior to data collection, the questions were pretested on 50 orphan children in order to assess the validity of the questionnaire.

Type III examination of subjects was conducted at respective orphanages. The data obtained were compiled systematically, transformed from a precoded proforma to a computer, and statistical analysis was done using Statistical Package for the Social Sciences (SPSS) version 15.0 statistical analysis software.

## RESULTS

A total of 1,375 subjects with mean age 10.23 ± 1.801 years were examined in the present study, out of which 964 (70%) were boys and 411 (30%) were girls. The study subjects were divided into three groups according to age: ≤6, 7 to 11, and ≥12 years. Highest number of subjects (976; 70.8%) were within the age group 7 to 11 years, which comprised of 743 boys (77.0%) and 233 girls (56.5%); 232 subjects (16.9%) were ≥12 years, which included 136 boys and 96 girls. The mean for years in institutionalization was 3.89 ± 0.523, 4.18 ± 0.539 for girls and 3.60 ± 0.513 for boys.

Table 1 presents the number of orphan children according to their age and the mean years of institution-alization with regard to their age and gender. The level of education was divided into three groups. Preschool group included study subjects before the start of formal schooling (KG, prenursery), which included 79 (5.72%) subjects. Primary level included subjects within 1st to 5th standard and comprised 1,070 subjects (77.64%), and Junior or above included subjects who were in 5th to 8th standard or above and comprised 226 subjects (16.35%).

Table 2 presents the caries prevalence in the primary dentition in the subjects within the age group ≤6 and 7 to 11 years. Caries prevalence was higher in subjects ≤6 years of age, presenting 50.9% caries prevalence, while subjects 7 to 11 years of age presented a much lesser caries prevalence of 25.2% in primary dentition. Male children presented higher caries prevalence in both the age groups in primary dentition. Significant differences were registered in caries prevalence between the age groups (p < 0.05).

Table 3 shows the caries prevalence in permanent dentition in the subjects within the age groups 7 to 11, which was 69.1%, while subjects ≥12 years presented 66.2%. The caries prevalence in these age groups did not show any significant difference (p = 0.540). Males presented higher caries prevalence in the age group of 7 to 11 years (69.8%), while females in the age group ≥12 years presented a higher caries prevalence (74.2%). The mean caries prevalence in the permanent dentition was 67.65%.

Table 4 presents the mean deft and defs in subjects ≤6 and 7 to 11 years in the primary dentition. The mean deft in subjects ≤6 years was 1.355 ± 1.79, while subjects 7 to 11 years presented lower mean deft of 1.03 ± 1.61 in comparison to the subjects of lesser age. The decayed component (dt) was found to be higher than other components in both the age groups. Significant differences were registered in mean dt and deft (p ≤ 0.05) in both the genders within the age group of ≤6 years, while in the age group of 7 to 11 years, significant difference was registered in all the components in primary dentition (p ≤ 0.05) within the genders and boys had higher dt than girls. Statistically significant difference was also recorded in between the two genders in defs (p ≤ 0.05).

**Table 1:** **Table 1:** Detailed frequency distribution of the study subjects in relation to age and years of institutionalization according to gender

				*Overall (n = 1375)*						*Male (n = 964 )*						*Female (n = 411)*					
*Sl. no.*		*Age in* *years*		*n*		*Mean years in* *orphanage*		*SD*		*n*		*Mean years in* *orphanage*		*SD*		*n*		*Mean years* *in orphanage*		*SD*	
1		6		46		3.23		1.48		23		3.26		1.57		23		3.2		1.41	
2		7		123		3.65		1.76		63		3.19		1.48		60		4.13		1.92	
3		8		107		3.64		2.05		51		3.10		2.20		56		4.14		1.77	
4		9		125		3.45		2.01		85		3.16		1.93		40		4.05		2.05	
5		10		464		4.14		2.34		365		4.10		2.29		99		4.27		2.13	
6		11		278		4.49		2.31		183		4.17		2.32		95		4.37		2.17	
7		12		232		4.67		2.40		194		4.24		2.47		38		5.11		2.58	
Mean						3.89 ± 0.523						3.60 ± 0.513						4.18 ± 0.539			

SD: Standard deviation

**Table 2:** **Table 2:** Frequency distribution of subjects, according to prevalence of dental caries in primary dentition (DMFT) in relation to age groups and gender

		*Total (n = 1,143)*						*Males (n = 828)*						*Females (n = 315)*					
*Age in* *years*		*Total*		*With caries*		*% prevalence*		*Total*		*With caries*		*% prevalence*		*Total*		*With caries*		*% prevalence*	
≤6		167		86		50.9		85		53		61.6		82		33		39.8	
7-11		976		246		25.2		743		196		26.3		233		35		15	
Total		1143		332		38.5		828		249		43.95		315		68		27.4	
χ^2^					53.43						62.931						22.198		
p					<0.001						<0.001						<0.001		

**Table 3:** **Table 3:** Frequency distribution of study subjects according to prevalence of dental caries in permanent dentition (DMFT) in relation to age groups and gender

		*Total (n = 1,208)*						*Males (n = 882)*						*Females (n = 329)*					
*Age in* *years*		*Total*		*With caries*		*% prevalence*		*Total*		*With caries*		*% prevalence*		*Total*		*With caries*		*% prevalence*	
7-11		976		676		69.1		745		520		69.8		233		156		66.7	
≥12		232		155		66.2		137		83		60.6		96		72		74.2	
Total		1208		831		67.65		882		603		65.2		329		228		70.45	
χ^2^					1.234						4.624						6.227		
^p^					0.540						0.099						0.044		

**Table 4:** **Table 4:** Dental caries experience in primary dentition (deft/ defs) of ≤6 and 7- to 11-year-old study subjects according to gender

		*Males* *(n = 85)*				*Females* *(n = 82)*							
*Variables*		*Mean*		*SD*		*Mean*		*SD*		*t-value*		*p-value*	
*≤6 years old*													
I. Teeth wise													
dt		1.63		1.86		0.65		1.25		3.991		<0.001	
et		0.20		0.94		0.19		0.63		0.040		0.969	
ft		0.01		0.11		0.02		0.15		0.611		0.542	
deft		1.84		2.14		0.87		1.44		3.450		0.001	
		1.355 ± 1.79											
II. Surface wise													
ds		2.20		2.62		0.86		1.70		3.937		<0.001	
es		0.99		4.72		0.94		3.11		0.079		0.937	
fs		0.01		0.11		0.05		0.31		1.026		0.307	
defs		3.20		5.70		1.84		3.62		1.836		0.068	
		2.52 ± 4.66											
*7-11 years*													
III. Teeth wise													
dt		1.01		1.69		0.46		1.03		2.814		0.005	
et		0.45		0.90		0.12		0.53		3.231		0.001	
ft		0.1		0.09		0		0		-		-	
deft		1.47		2.05		0.59		1.17		3.811		<0.001	
		1.03 ± 1.61											
IV. Surface wise													
ds		1.42		2.51		0.56		1.27		3.134		0.002	
m/e		2.03		3.99		0.58		2.59		3.142		0.002	
fs		0.04		0.38		0		0		-		-	
defs		3.50		5.32		1.13		2.95		3.960		<0.001	
		2.31 ± 4.13											

SD: Standard deviation

**Table 5:** **Table 5:** Mean dental caries experience in permanent (DMFT/ DMFS) dentitions in study subjects 7 to 11 and ≥12 years of age, according to gender

		*Males* *(n = 743)*				*Females* *(n = 233)*							
*Variables*		*Mean*		*SD*		*Mean*		*SD*		*t-value*		*p-value*	
*7-11 years*													
I. Teeth wise													
DT		1.14		1.35		1.33		1.53		-1.007		0.315	
MT		0.10		0.41		0.54		1.23		-3.849		0.000	
FT		0.01		0.12		0.08		0.42		-1.820		0.070	
DMFT		1.30		1.49		1.82		2.22		-2.170		0.031	
Mean		1.56 ± 1.85											
II. Surface wise													
DS		1.31		1.71		1.69		1.95		1.563		0.119	
MS		0.43		1.86		1.67		5.13		2.600		0.010	
FS		0.01		0.12		0.13		0.66		2.085		0.038	
DMFS		1.82		2.60		3.49		5.88		2.958		0.003	
Mean		2.65 ± 4.24											
*≥12 years old*													
III. Teeth wise													
DT		1.43		1.85		1.61		1.58		-1.358		0.175	
MT		0.15		0.53		0.17		0.78		-0.366		0.715	
FT		0.07		0.34		0.06		0.29		0.266		0.790	
DMFT		1.64		1.97		1.85		1.88		-1.393		0.164	
Mean		1.74 ± 1.92											
IV. Surface wise													
DS		1.58		1.68		1.74		1.86		-1.238		0.216	
MS		0.76		2.62		0.63		2.55		0.637		0.524	
FS		0.09		0.43		0.08		0.37		0.369		0.712	
DMFS		2.41		3.19		2.46		3.24		-0.188		0.851	
*Mean*		2.43 ± 3.21											

SD: Standard deviation

Table 5 presents the mean DEFT and DEFS in subjects 7 to 11 and ≤12 years in the permanent dentition. Mean DMFT for subjects 7 to 11 years was 1.56 ± 1.85, while for subjects ≥12 years it was 1.74 ± 1.92, which was more than the latter age group. In permanent dentition, higher caries experience was seen in girls as compared with the boys, while statistically significant difference in various components was only found in the age group of 7 to 11 years (p = 0.031).

Table 6 presents the relation between the reported oral hygiene practices and caries prevalence in orphan children. No significant difference was seen in any of the responses in between the genders. Highest caries prevalence was seen in the subjects using datun sticks as a method to clean their teeth (80.5%), while use of toothbrush was the most prevalent method of cleaning the teeth in both the genders. Toothpaste was the widespread material used followed by toothpowder; caries prevalence in subjects using these was 61.1 and 65.8% respectively. Subjects who reported that they were cleaning their teeth occasionally were having the higher caries prevalence of 88.9%, whereas subjects who reported that they never cleaned their teeth had a caries prevalence of 92.8%. Out of the total population of 880 subjects using toothbrushes, replacement periods of the toothbrush varied, while caries prevalence of the subjects never replacing their teeth and who did not remember was highest with 77.2%. Regarding the visits to dentist, 32.84% had visited a dentist more than a year ago, while 31.57% had never visited a dentist and only 17.42% reported having regular dental checkups (not included in table).

Highest number of subjects, i.e., 689 (50.1%), reported using toothpaste having fluoride incorporated in it, while 447 subjects (32.5%) reported using nonfluoridated toothpaste. In the studied population, 85.79% reported taking sweets occasionally, and only 12.22% subjects specified taking sweets about one to three times per day.

## DISCUSSION

In India, many epidemiological studies on oral health in privileged children have been carried out, while such studies in orphan children are scarce and none has been done in Jammu and Kashmir state. Thus, the present descriptive study was undertaken to determine the dental caries status of institutionalized orphan children from Jammu and Kashmir, India.

**Table 6:** **Table 6:** Caries prevalence of DMFT and deft (for ≤6-year-olds only) of the study subjects according to practices, in relation to gender

		*Total*						*Males*						*Females*					
*Practice*		*Total*		*With caries*		*% prevalence*		*Total*		*With caries*		*% prevalence*		*Total*		*With caries*		*% prevalence*	
I. Method of cleaning																			
Toothbrush		880		668		75.9		593		462		77.9		287		206		71.7	
Finger		59		42		71.1		33		26		78.7		26		16		61.5	
Datun stick		416		335		80.5		322		260		80.7		94		75		79.7	
None		20		16		80		16		13		81.2		4		4		100	
χ^2^					5.929						1.579						6.799		
p					0.115						0.664						0.079		
II. Material used for teeth cleaning																			
Salt and oil		1		1		100		1		1		100		0		0		0	
Toothpaste		1052		643		61.1		724		437		60.3		328		206		62.8	
Coal		0		0		0		0		0		0		0		0		0	
Toothpowder		322		212		65.8		239		156		65.2		83		56		67.4	
χ^2^					0.361						1.251						1.03		
p					0.635						0.535						0.31		
III. Frequency of cleaning																			
Never		42		39		92.8		37		34		91.8		5		5		100	
Once		700		530		75.7		477		368		77.1		223		162		72.6	
Twice		377		294		77.9		276		221		80.0		101		73		72.2	
More than twice		47		33		70.2		41		29		70.7		6		4		66.6	
Occasionally		209		186		88.9		133		123		92.4		76		63		82.8	
χ^2^					4.693						3.628						3.216		
p					0.320						0.459						0.522		
IV. How often do you replace your toothbrush?																			
1-3 months		272		193		70.9		131		93		70.8		141		100		71	
4-6 months		127		97		76.5		111		85		76.6		16		12		75	
7-12 months		38		27		71.1		25		18		71.7		13		9		70	
More than one year		95		70		74.3		78		57		73.6		17		13		78.2	
Don’t remember		15		12		77.2		15		12		77.2		0		0		0	
Never		333		257		77.2		233		181		77.4		100		76		76.6	
χ^2^					6.212						7.530						18.653		
p					0.286						0.184						0.124		
When did you last visit a dentist?																			
One year ago		441		333		75.5		298		224		75.2		143		109		76.2	
More than one year		213		174		81.6		109		94		78.8		94		80		85.1	
Only when in pain		31		24		77.4		26		20		76.9		5		4		80.0	
Regular check-ups		234		190		81.2		201		170		84.6		33		20		60.6	
Never		424		320		75.5		302		245		81.1		122		75		61.5	
Total		1343		1041		77.5		946		753		79.6		397		299		72.5	
χ^2^					4.693						1.354						1.003		
p					0.286						0.184						0.002		

In the present study, subjects were divided into three groups according to age: ≤6, 7 to 11, and ≥12 years. Girls constituted only 30%, which can be due to the fact that given the stigma of orphaned life, girls prefer to live with relatives or work as housemaids for safety and security.^12^

The total caries prevalence in deciduous dentition of study subjects ≤6 years of age in the present study was 51.9%, while higher caries prevalence of 90% has been reported in primary dentition in a similar population.^13^ Previously, Bali et al^14^ have reported that caries prevalence of Indian children 5 years of age was 50%, while in Jammu and Kashmir state, it was 50.9%; moreover, similar caries prevalence has been reported earlier in privileged child populations around the worls.^15,16^ The mean recorded deft of subjects ≤6 years of age was 1.355 ± 1.79, while low caries (0.70 deft) was demonstrated by institutionalized street children of Andhra Pradesh^17^ and orphans in Mashad, Iran.^18^ On the contrary, higher deft was recorded in orphan children in Pune^19^ and Mexico.^13^

The caries prevalence for primary dentition for age group 7 to 11 years was found to be 25.2%, and mean deft was 1.03 ± 1.61; these findings are similar to those found in Mexican schoolchildren.^20^ However, much higher caries prevalence in similar age group has been reported from Maharashtra,^21^ Orissa,^22^ and other parts of the country in privileged child populations.^23^

Moreover, the mean deft recorded was less in girls ≤6 years of age (0.87 ± 1.44) as well as 7 to 11 years of age (0.59 ± 1.17), which is similar to the results of the National Oral Health Survey and fluoride mapping in Jammu and Kashmir state during 2002-2003^14^ and other previous studies.^17^ On the contrary, girls in Mashhad orphanages had higher dmft than boys.^24^ The results of the present study demonstrate a similar caries prevalence in children ≤6 years, as seen in nonorphan population in the same state,^14^ which could be explained by their least stay in institutionalized conditions by this age group; thus, a similar oral hygiene attitude as of privileged children can be seen in these children. The decrease in caries prevalence in primary dentition with increasing age, i.e., from ≤6 to 7 to 11 years, can be because of the natural exfoliation of deciduous teeth.^18^ As the tooth erupts into the oral cavity because of posteruptive maturation, tooth becomes resistant to caries.^21^

The overall caries prevalence for permanent dentition was 67.65% in children aged 7 to 11 years and 66.2% for more than ≥12-year-old subjects. These results are similar to Romanian orphaned and abandoned children^25^ and caries prevalence in some privileged children popula-tion,^22^ while lower caries prevalence has been reported for 12-year-old children in Jammu and Kashmir state (47.5%) as well as the whole of India (52.5%).^14,22,26^

Mean DMFT for children 7 to 11 years was similar to subjects from Mashad^24^ and child populations in many third world countries.^16,27^ Mean DMFT among >12-year-old children is in accordance with caries in 12-year-old children of the whole of India (1.7 DMFT)^14^ and other countries.^28^ However, much higher mean DMFT has been reported in 12-year-olds elsewhere.^20,27^ Furthermore, the results from the present study depict that the caries experience was largely made by decayed (d/D) component in both primary and permanent dentition. These findings suggest lack of dental care in these groups. These findings have been reported earlier also.^29^

In permanent dentition, orphan girls presented higher caries than orphan boys, which is in contrast to findings in privileged children as reported in the National Oral Health Survey 2002-2003, Jammu and Kashmir.^14^ Orphan girls carry bigger burden of dental caries and that could be explained by their easy access to food supplies and their frequent snacking during food preparation.^30^ The higher caries in the permanent dentition can also be because 30.23% subjects reported using miswak sticks, as previously reported for children population in the National Oral Health Survey 2002-2003 in Jammu and Kashmir.^14^ Previously, it has been reported that in Muslim religious institutions, children are taught to use miswak at about age 6.^31^

The data on oral health behavior collected from all of the subjects were done by questionnaire adopted from Al-Omiri et al,^11^ which was pretested before the study was conducted, assuring that technical jargon was avoided and the language level was set to allow proper comprehension by the subjects.

The information regarding the usage of oral hygiene aids showed similarity to the results of many previous studies done in institutionalized children who reported regular toothbrushing and use of toothpaste^12,17,32,33^ and is also similar to results from the National Oral Health Survey, Jammu and Kashmir state.^14^ The response to the frequency of cleaning the teeth was similar as reported earlier from orphanages in Mexico City^13^ and South Indian children who were staying in ashrams.^33^ On the contrary findings from Udaipur orphanage^32^ institutionalized street children in Jordan^12^ and data from the National Oral Health Survey India and Jammu and Kashmir state^14^ show lesser frequency of cleaning he teeth than seen in this population.^14^ The widespread use of toothbrush in the subjects can be because various charity organizations provide oral education and oral health care products to orphanages. It was also seen that subjects cleaning their teeth twice in a day with toothbrush and toothpaste exhibited more caries prevalence (75%); this can be attributed to the fact that many children lack the knowledge of proper toothbrushing,^34^ and brushing the teeth very fast and with an improper technique greatly reduces its efficacy in reducing dental disease.^35^

Regarding the visit to a dentist, 31.57% subjects had never visited a dentist. These findings are similar to the findings of national data for the state.^14^ The reason can be limited resources as well as dental health personnel and the attitude of these children toward dental professionals. Furthermore, children might be satisfied with the status of their teeth and thus do not recognize the need for regular dental visits.^36^

Regarding the replacement of the toothbrushes, 77.2% never replaced their toothbrushes. On the contrary, national data from the same state shows that 67.7% of 5-year-old and 72.7% of 12-year-old privileged children replaced their toothbrushes within 1 to 3 months.^14^ This may be explained by the fact that cost of replacing toothbrushes at frequent intervals may be prohibitive in institutions harboring large number of orphan children which may be due to. In the present study, 59.01% of the subjects reported knowledge of fluoride in the toothpaste; these findings are similar to the findings in Dar-es-Salaam of institutionalized former street children^12^ and to many previous studies.^8,11^ The higher use of fluoridated toothpaste could be because of its availability as the World Health Organization (WHO) has recommended to make fluoridated toothpaste affordable and available in developing countries.^37^

The data collected for habits and practices may have certain limitations. With regard to oral hygiene habits and practices and frequency of dental visits, courtesy bias and overreporting have to be assumed, whereas for the consumption of sugary foods and drinks, we assume a central tendency bias and may have been probably under-reported. In addition, recall bias and social desirability bias could be considered with respect to consumption of food items and frequency of toothbrush replacement. Moreover, the data on oral health knowledge, attitudes, and practices of orphan children are very scarce. Due to this fact, comparison with similar population has not been possible, and a comparison with a nonorphan population shall remain a limitation.

## CONCLUSION

It can be concluded that conditions and experiences early in life leave an indelible imprint on the individual. The negative impact of institutionalization on a young growing person and nonrecognition of the child as a “person” and “rights holder” have enormous impact on the development of children. The results of the present study show that the caries level in orphan children studied is more than the WHO (Oral Health Goals 2010) target of mean DMFT/dmft of 1.5.^38^ Also, major difference existed between decayed (dt/DT) and filled (ft/FT) components, which indicate poor practices and professional services to this study group.

## RECOMMENDATIONS

These extensive unmet dental needs of this socially disabled strata should be promptly met by the health care providers to facilitate better health in these deprived children. In order to improve the dental health status of this underprivileged orphan population, based on our results, we propose that a preventive program can be planned and implemented on these factors: (A) In general, we should change the perceptions of caregivers and orphaned about oral health. (B) We should provide oral health education. (C) Caries prevention programs as placing sealants and fluoride programs shall be started for such disadvantaged children. (D) School oral health services should be provided.

Clearly, a single individual has least control over such requirements, and these mostly or entirely can be improved at the population or group level.
